# Ureteral frozen section analysis during radical cystectomy: Do margins matter?

**DOI:** 10.4103/0970-1591.32082

**Published:** 2007

**Authors:** Rajiv Mukha, Santosh Kumar, Nitin S. Kekre

**Affiliations:** Department of Urology, Christian Medical College, Vellore, Tamilnadu, India

One of the objectives of extirpative oncological surgery is negative surgical margins. The gold standard of treatment of high-grade recurrent superficial bladder carcinoma and muscle-invasive organ-confined bladder carcinoma is radical cystectomy. Transitional cell carcinoma has always been associated with a pan urothelial malignant field change. Frozen section analysis (FSA) to assess negative ureteral margins has been reported from as early as the late 1960s and early 1970s.[1–4] Based on these findings, FSA of the ureteral margins was recommended at cystectomy. It has been suggested that this procedure reduces the risk of missing important pathological evidence. In cases of pathological abnormalities at the ureteral end, a more proximal ureteral segment can be excised to ensure a tumor-free ureterointestinal anastomosis.[5] An FSA for ureteral margins involves an additional cost, increase in the operating and anesthesia time leading to an added burden on the health resources and the patient. According to a recent report,[4] FSA has sensitivity as low as 45% but a high positive predictive value of 81%. In this review, we review the evidence regarding the usefulness of FSA in radical cystectomy.

## THE EVIDENCE

The distal ureter has the highest incidence of urothelial malignancy in patients with carcinoma *in situ* (CIS) of the urinary bladder.[6] The range has been reported to be as high as 8-18% in patients with carcinoma *in situ* or solid tumors undergoing radical cystectomy for TCC with coexisting CIS in the bladder.[1,4] Local recurrences have been reported at the site of the ureteroenteric anastomosis and have the potential to be a major morbidity.[7] Carcinoma *in situ* of the ureteral margin has been discovered in up to 8.5% of patients undergoing radical cystectomy. Positive ureteral margin CIS has been associated with a high risk of upper tract recurrence, with CIS of the ureteral margins having a risk of 17% and negative histology of the ureteral margin having a 3% upper tract recurrence risk.[5] Up to 8.3% of patients can have pathological abnormality on a ureteral margin FSA.[4] Cooper *et al* suggested that FSA of ureteral margins enables a more thorough removal of malignant urothelium. Frozen section analysis of the ureteral margins has been recommended by the European Association of Urology and the National Comprehensive Cancer Network for patients undergoing radical cystectomy with extensive CIS of the bladder. Ureteral CIS has most commonly been observed in patients with multifocal, high-stage, high-grade tumors and tumors involving the prostatic urethra.[8–9]

With the above evidence it would seem justifiable to perform an FSA of all ureteral margins. However, evidence to the contrary is just as extensive and impressive. Sharma *et al* [1] discovered CIS on the final pathological report of 17 in a series of 205 patients examined. Only one of the 17 had ureteroileal recurrence on follow-up. In a follow-up of the same series Linker and Whitmore[10] suggested that ureteral CIS had little overall effect on the clinical outcome of the patients studied and that a conservative approach with a clinical follow-up was probably most appropriate for these patients. Similar outcomes were described by Johnson *et al*[9] in their series of 403 cystectomies. They reported an unsuspected malignant ureteral margin in 2% (eight patients). Only one had clinically evident disease on a six-year follow-up. Upper tract recurrences but not anastomotic recurrences have been reported to be higher in patients with evidence of the involvement of the ureter.[11] Upper tract recurrences have been reported to have a risk as high as 17% with negative distal ureters having a 3% risk. However it must be kept in mind that the majority of these patients succumb to systemic metastatic disease. In a series of 1330 radical cystectomies, Ganesh *et al*[11] found ureteral involvement in 9% at the time of surgery. They calculated a five-year probability of upper tract recurrences as 13%. Evidence of ureteral margin involvement was associated with a higher likelihood of upper tract recurrence but not anastomotic recurrences or overall survival. The American Society of Oncology[12] looked at the significance of ureteral sampling at radical cystectomy in 2005. Of the 966 patients studied 13% had ureteral abnormalities on the final pathological analysis. They, however, concluded that ureteral margin sampling if applied to all patients undergoing radical cystectomy had questionable value in predicting upper tract recurrences. They suggested the need to identify the subset of population benefiting the most from the FSAs. Sequential sectioning of the ureters to reach a negative margin does not eliminate the risk of anastomotic or upper tract recurrence,[11] making such an exercise seem futile. TCC being a panurothelial change may have skip lesions, making the practice of a disease-free margin seem meaningless.

It would seem reasonable to do FSA on palpable induration or frank tumor infiltration of the distal ureter discovered unexpectedly at the time of the operation. Similarly, in those with diffuse CIS the risk of ureteral involvement may be greater than average necessitating the need for an FSA. But should one do an FSA on patients with prostatic TCC with the increased risk of metastatic disease? In this subset the mainstay of treatment is effective chemotherapy which provides a greater survival benefit than the absolute eradication of distal ureteral atypia.[8] An FSA is also fraught with the risks of false negative and positives. In the most recent analysis on ureteral margins and FSA, Osman *et al*[4] found 13 out of 29 true positives with 16 of 29 false negatives. Thus reflecting the limited margin of safety with frozen sections. Frozen section analysis in a low turnover center may have a higher number of pathologist-related misinterpretations.

## CONCLUSION

The presence of positive CIS at the ureteral margin predicts risk of upper tract recurrence and not necessarily anastomotic recurrence. Routine FSA of the ureteral margins is probably unnecessary in the majority of patients undergoing radical cystectomy. In patients with high risk of upper tract recurrence, such as diffuse CIS or induration at the margins a frozen section analysis of the ureteral margins may be helpful in tailoring the operation. However, the practice of resecting the ureter to achieve a negative margin does not seem to prevent upper tract recurrence.
